# Characterization of a Novel Alginate Lyase from Marine Bacterium *Vibrio furnissii* H1

**DOI:** 10.3390/md16010030

**Published:** 2018-01-15

**Authors:** Xiaoyan Zhu, Xiangqian Li, Hao Shi, Jia Zhou, Zhongbiao Tan, Mengdi Yuan, Peng Yao, Xiaoyan Liu

**Affiliations:** 1 Jiangsu Provincial Engineering Laboratory for Biomass Conversion and Process Integration, Huaiyin Institute of Technology, Huaian 223003, China; lixq@hyit.edu.cn (X.L.); ilyshihao@163.com (H.S.); jiazhou@hyit.edu.cn (J.Z.); tanzb@hyit.edu.cn (Z.T.); 2 School of Life Science and Food Engineering, Huaiyin Institute of Technology, Huaian 223003, China; 15996162102@163.com (M.Y.); yaopeng0224@163.com (P.Y.); 3 Jiangsu Key Laboratory for Biomass-based Energy and Enzyme Technology, Huaiyin Normal University, Huaian 223300, China; catty5082003@163.com

**Keywords:** alginate lyase, *Vibrio furnissi*, purification, characterization, oligosaccharides, polysaccharide lyase family 7

## Abstract

Alginate lyases show great potential for industrial and medicinal applications, especially as an attractive biocatalyst for the production of oligosaccharides with special bioactivities. A novel alginate lyase, AlyH1, from the marine bacterium *Vibrio furnissii* H1, which has been newly isolated from rotten seaweed, was purified and characterized. The purified enzyme showed the specific activity of 2.40 U/mg. Its molecular mass was 35.8 kDa. The optimal temperature and pH were 40 °C and pH 7.5, respectively. AlyH1 maintained stability at neutral pH (7.0–8.0) and temperatures below 30 °C. Metal ions Na^+^, Mg^2+^, and K^+^ increased the activity of the enzyme. With sodium alginate as the substrate, the *K*m and *V*max values of AlyH1 were 2.28 mg/mL and 2.81 U/mg, respectively. AlyH1 exhibited activities towards both polyguluronate and polymannuronate, and preferentially degraded polyguluronate. Products prepared from sodium alginate by AlyH1 were displayed to be di-, tri-, and tetra-alginate oligosaccharides. A partial amino acid sequence (190 aa) of AlyH1 analysis suggested that AlyH1 was an alginate lyase of polysaccharide lyase family 7. The sequence showed less than 77% identity to the reported alginate lyases. These data demonstrated that AlyH1 could be as a novel and potential candidate in application of alginate oligosaccharides production with low polymerization degrees.

## 1. Introduction

Alginate is a major component in the cell walls of marine macroalgae, which are available in large amounts in marine environments and are one of the fastest growing organisms in the world [1]. It is a linear acidic polysaccharide consisting of 1,4-linked β-d-mannuronate and α-l-guluronate residues, arranged in varying sequences with uniform regions of mannuronate (poly-M) or guluronate (poly-G), or a mixture of mannuronate and guluronate (poly-MG) [2,3]. 

Alginate can be depolymerized into alginate oligosaccharides. Compared with corresponding chemical and physical methods, enzymatic depolymerization has certain advantages, including high production yields and the production of specific oligosaccharides [4]. Alginate lyase degrades alginate via the β-elimination mechanism, producing various oligosaccharides with unsaturated uronic acid at the non-reducing terminus and unsaturated uronic acid monomers [5]. Based on the substrate specificities, alginate lyases are classified into mannuronate lyase (EC 4.2.2.3) and guluronate lyase (EC 4.2.2.11) that preferentially break up the M- and G- rich alginates, respectively [1]. Some alginate lyases show bifunctional activity for both poly-M and poly-G [6,7]. Alginate lyases are also characterized by their structure, termed as polysaccharide lyase (PL) families. On the basis of alginate lyase primary structures, alginate lyases are organized into seven PL families including PL5, PL6, PL7, PL14, PL15, PL17, and PL18 [2,8]. Alginate lyases have attracted increasing attention in recent years due to their critical role in the biotransformation of alginate into high-value and functional oligosaccharides that exhibit a variety of biological activities such as antitumor [9], non-specific immunostimulatory [10], and antioxidant activity [11], as well as root growth-promoting activity towards plants [12]. Alginate lyases also play an important role in the elucidation of alginate fine structures [13], the protoplast preparation of red and brown algae [14], and the treatment of cystic fibrosis [15], as well as biofuel production [3]. 

Various alginate lyase-producing organisms, including bacteria, fungi, brown algae, marine mollusks, and viruses, have been explored and the enzyme characterization investigated [5,16]. In this study, we report on a new alginate lyase-producing bacterium; the alginate lyase, AlyH1, was purified and its enzymatic properties were investigated, and its partial amino acid sequence was determined. 

## 2. Results and Discussion

### 2.1. Isolation of Alginate-Degrading Microorganism

A large number of alginates are annually produced by algae in the sea. Various alginate lyases produced by marine microorganisms are actively involved in marine alginate degradation. Utilizing sodium alginate as the sole carbon source, this work isolated several microbial strains based on the appearance of the clearing hydrolytic zone and alginate lyase activities from the fermentation culture. The strain with the most remarkable activity was selected and designated as H1 for further studies. A 1475 bp fragment of the 16S rRNA gene of the strain H1 (Genbank accession number: MG214325) was cloned and sequenced. The alignment results revealed that 16S rRNA gene sequences of strain H1 was 100% identical to the sequence of *Vibrio furnissii* NCTC 11218 chromosome 1 (CP002377.1) and 99% identical to the 16S rRNA gene sequence of *V. furnissii* JCM 1282 (LC050179). According to the blast result and phylogenetic position of its 16S rRNA (Figure S1), H1 was assigned to the genus *V. furnissii* and designated as *V. furnissii* H1. Several papers had previously reported that the *Vibrio* sp. could produce alginate lyase (Table 1). To the best of our knowledge, this study is the first to report on alginate lyase derived from *V. furnissii*. 

### 2.2. Purification of AlyH1

**Figure 1:**
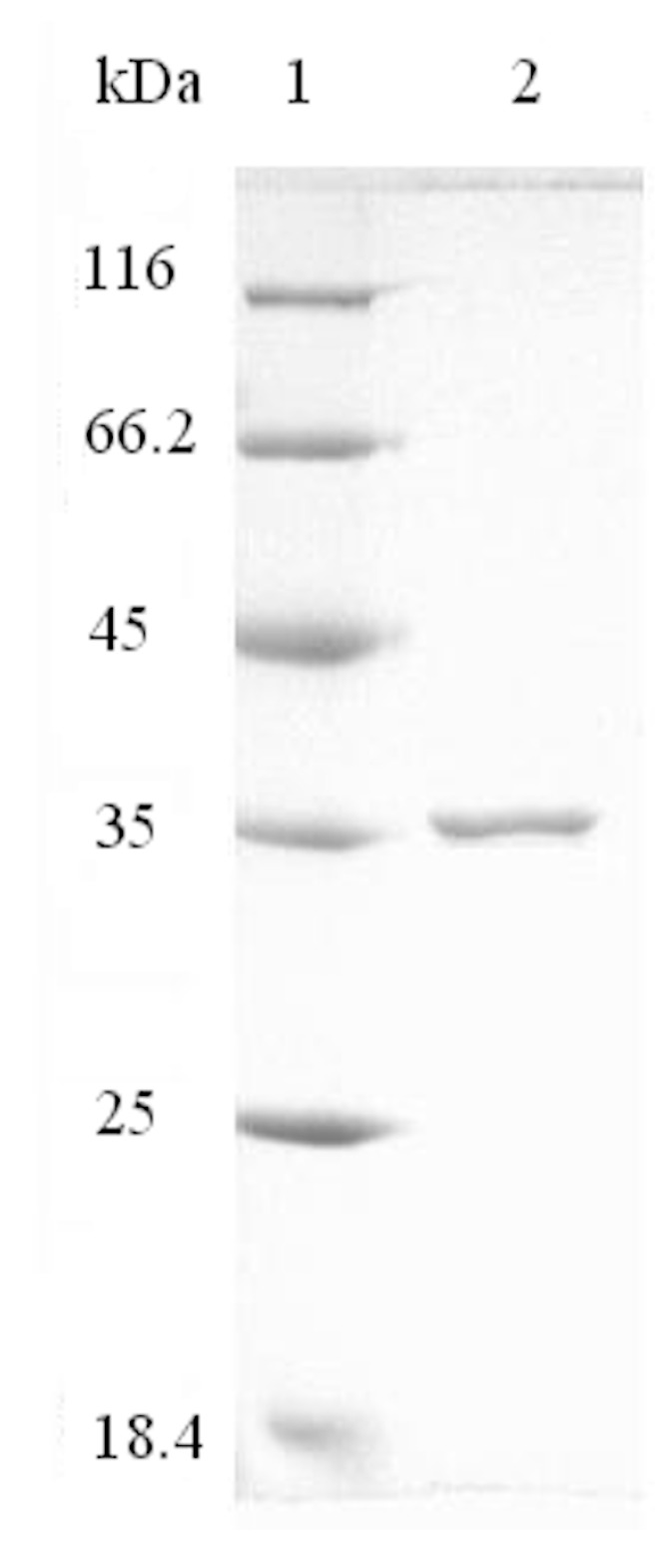
SDS-PAGE of AlyH1 (lane 2). The marker proteins (lane 1) are commercially-obtained standards as described.

AlyH1 was purified by ammonium sulphate fractionation and two-step column chromatographic procedures with a final yield of 9.28% (Table 2). An approximately 18.46-fold purification was obtained with a specific activity of 2.40 U/mg for purified AlyH1. The purified enzyme showed a single band on sodium dodecyl sulfatepolyacrylamide gel electrophoresis (SDS-PAGE) and the molecular weight of the enzyme was estimated to be 35.8 kDa (Figure 1). 

### 2.3. Characterization of AlyH1 

#### 2.3.1. Effects of Temperature and pH on AlyH1 Activity and Stability

**Figure 2:**
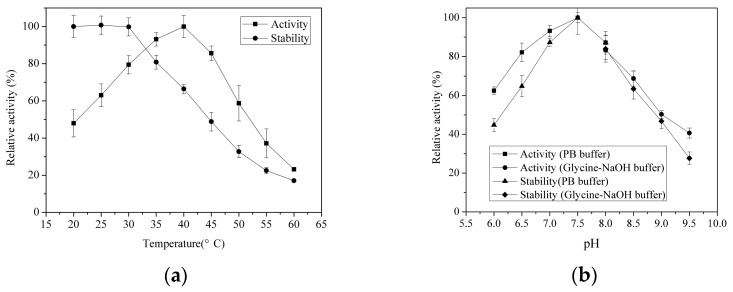
Effects of temperature and pH on the activity and stability of AlyH1. (**a**) Temperature profiles. The effect of temperature on AlyH1 activity was measured at different temperatures (20–60 °C) in 50 mM sodium phosphate buffer (PB, pH 7.5); the activity at 40 °C was taken as 100%. Thermal stability of AlyH1 was determined at 40 °C in 50 mM PB buffer (pH 7.5) after the purified enzyme solution was incubated at different temperatures for 30 min; the residual activity at 20 °C was taken as 100%. (**b**) pH profiles. The activity was measured at 40 °C in PB buffer (pH 6.0–8.0) and glycine-NaOH buffer (pH 8.0–9.5); the activity at pH 7.5 was taken as 100%. The pH stability was determined at 40 °C in 50 mM PB buffer (pH 7.5) after the purified enzyme solution was incubated at 4°C in PB buffer (pH 6.0–8.0) and glycine-NaOH buffer (pH 8.0–9.5) for 12 h; the residual activity at 7.5 °C was taken as 100%.

Optimal temperature of the alginate lyases from marine bacteria differed significantly. The optimal temperatures of the alginate lyases from *Vibrio* sp. were 16–40 °C (Table 1). As shown in Figure 2a, AlyH1 had maximum activity at 40 °C, which was higher than most other alginate lyases from *Vibrio* sp. AlyH1 exhibited an excellent stability below 30 °C. More than 60% of residual activity was still maintained after incubation at 40 °C for 30 min. Zhu et al. recently reported an alginate lyase (AlgNJU-03) from *Vibrio* sp. NJU-03 possessed approximately 40% activity after incubation at 40 °C for 30 min [19]. The optimal pH of the enzyme was found to be 7.5 (Figure 2b). The optimal pHs for most alginate lyases from marine bacteria are between 7.0 and 8.5, and AlyH1 corresponded with this range. After incubation for 12 h, AlyH1 retained more than 60% activity in a pH range of 6.5 to 8.5 and 80% activity in a pH range of 7.0 to 8.0. The best stability appeared at pH 7.5 (Figure 2b). Thus, the neutral pH condition was proven to be suitable for AlyH1 to conduct catalysis reaction and maintain activity. 

#### 2.3.2. Effects of NaCl Concentration and Metal Ions on AlyH1 Activity

As the alginate lyase was from a marine bacterium, the effect of NaCl in different concentrations (0–0.6 M) on the activity of AlyH1 was investigated. AlyH1, showing the highest activity in the presence of 0.3 M NaCl, had around 23% more activity than the control without the addition of NaCl (Figure S2a). This indicated that NaCl acted as a strong activator for this alginate lyase. A similar phenomenon was observed with the alginate lyase from *Vibrio* sp. strains such as *Vibrio* sp. QY105, *Vibrio* sp. YKW-34 and *Vibrio* sp. SY08, but the optimal concentration of NaCl was different from each other [17,24,25]. Little enzymatic activity of alginate lyase (AlyV5) from *Vibrio* sp. QY105 was detected without NaCl and the highest activity was observed in the presence of 0.5 M NaCl [24], while the activity of alginate lyase from *Vibrio* sp. YKW-34 was completely lost by dialysis and restored by addition of NaCl, and the optimal activity exhibited in 0.1 M NaCl [25]. However, the alginate lyase from *Vibrio* sp. SY08, the enzyme was active in absence of NaCl and the highest activity was observed in the presence of NaCl less than 0.1 M [17].

The effects of metal ions on AlyH1 activity were investigated by using various metal ions (Figure S2b). The enzyme activity was inhibited by Zn^2+^, Fe^2+^, Cu^2+^, Mn^2+^, and Ag^+^. However, K^+^ and Mg^2+^ displayed activating effects among the metal ions investigated. Mg^2+^ showed the most stimulating effect with 119.25% of relative activity followed by K^+^ with 110.31%. The importance of Mg^2+^ and K^+^ for some alginate lyases from marine bacteria such as *I. halotolerans* CGMCC 5336 [6] and *Cobetia* sp. WG-007 [32], has also been reported. 

#### 2.3.3. Kinetic Parameters 

The Lineweaver-Burke graph demonstrated that the *K*m and *V*max values of AlyH1 using sodium alginate as the substrate were 2.28 mg/mL and 2.81 U/mg, respectively. The *K*m value of alginate lyase from *Cobetia* sp. WG-007 [32] and *Vibrio sp*. YWA [26], however, was 2.80 and 72.73 mg/mL toward sodium alginate, respectively. These values suggest that AlyH1 had a high affinity and catalytic efficiency for the sodium alginate substrate. 

#### 2.3.4. Substrate Specificity

The substrate specificity of AlyH1 was characterized. The results showed that AlyH1 was active in degrading both poly-M and poly-G, indicating that it is a bifunctional alginate lyase. The relative activities of sodium alginate, poly-G and poly-M were 100.00 ± 5.76%, 128.37 ± 4.35%, and 52.14 ± 3.52%, respectively. It preferred poly-G over poly-M. Alginate lyases from different organisms showed differences in specific activity. The alginate lyases found in *Pseudoalteromonas* sp. SM0524 [33], *Aspergillus oryzae* [34], and *Vibrio* sp. YKW-34 [25] preferred to hydrolyze poly-M rather than poly-G, and alginate lyase from *Agarivorans* sp. JAM-A1m [35] and *Vibrio* sp.NJU-03 [19] preferred to degrade poly-G rather than poly-M, while some alginate lyases only showed the activity towards poly-G or poly-M [18,23]. 

#### 2.3.5. Thin-Layer Chromatography Analysis of the Degradation Products

**Figure 3:**
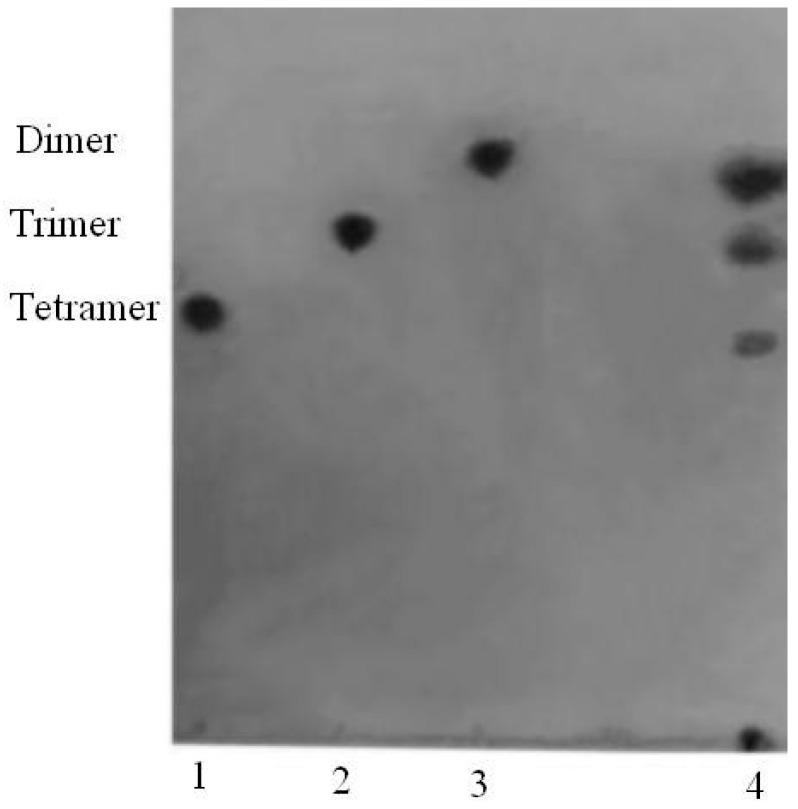
TLC analysis of the degradation products of sodium alginate. A 1 mL reaction mixture containing 1.0% sodium alginate was incubated at 30 °C for 24 h. The reaction products were separated on a TLC plate with 1-butanol: acetic acid: water (3:2:2, *v*/*v*) and visualized with 10% (*v*/*v*) sulphuric acid in ethanol. Lane 1: tetramer; Lane 2: trimer; Lane 3: dimer; Lane 4: degraded sodium alginate (1.0%).

The degradation products of the sodium alginate by AlyH1 were analysed by thin-layer chromatography (TLC). The results on the TLC plates clearly indicated that di-, tri-, and tetra-alginate oligosaccharides were generated (Figure 3). Different kinds of oligosaccharides were released by alginate lyases. The alginate lyase from *Vibrio* sp. W13 and *Microbulbifer* sp. 6532A produced oligosaccharides with degrees of polymerization of 2–6 [20,36]. The commercial enzyme originated from *Flavobacterium* sp. degraded alginate into penta- to hepta-oligosaccharides [37]. It indicated that AlyH1 may be a good tool for preparation of lower molecular weight alginate products than the commercial enzyme. 

#### 2.3.6. Determination of Partial Amino Acid Sequences of AlyH1

An internal peptide sequence KDNVMHLTFKK of AlyH1 was determined. The sequence was blasted at the National Centre for Biotechnology Information databases (NCBI) database. It was completely identical to PL 7 family protein from *Vibrio* sp. CA-1004 (WP_086981048.1) and 90% identical to PL 7 family protein from *V. rumoiensis* (WP_017024406.1). 

To obtain more information from AlyH1, degenerate polymerase chain reaction (PCR) was carried out. AlyH1 mainly hydrolysed poly-G and the identified peptide showed high identity to a PL 7 family protein. It was speculated that AlyH1 was a PL7 alginate lyase. PL7 alginate lyase contain three highly conserved regions, I R(S/N) ELR(E/A/V) (M/T/Q), II Q (I/V) H, III YFKAG(N/L/A/V) Y [38]. Therefore, degenerate primers Valg1 and Valg2 were designed corresponding to conserved regions I and II. With the degenerate PCR, the partial nucleotide sequence (570 bp) of alyH1 (AlyH1 gene) was identified (Figure S3). The sequence was blasted in NCBI, and results showed that this nucleotide sequence displayed the highest identity of 67% to *Agarivorans* sp. L11 alkaline alginate lyase (KM018274.1) with the BLAST algorithm of discontiguous megablast or blastn, while no significant similarity was found with the BLAST algorithm of megablast. It means that the identified partial sequence of alyH1 had the low homology to the reported nucleotide sequence of alginate lyases. Efforts are underway to obtain complete sequence of alyH1.

**Figure 4:**
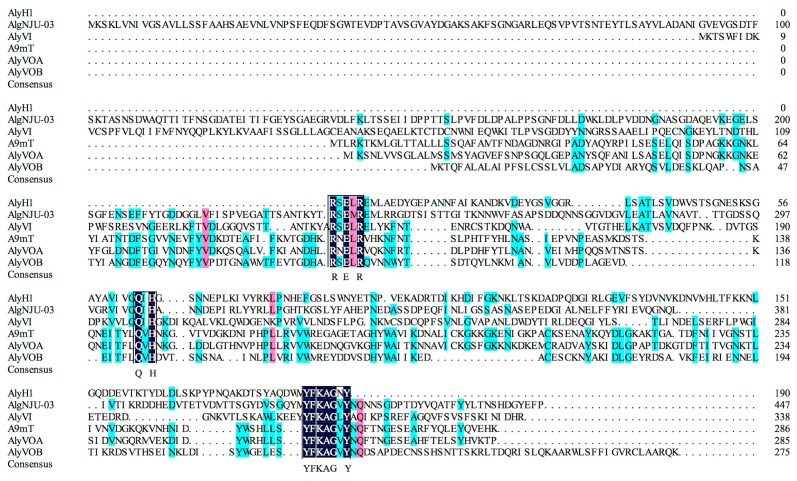
Comparison of the partial amino acid sequence of AlyH1 with alginate lyases AlgNJU-03 from *Vibrio* sp. NJU-03 (ASA33933.1), AlyVI from *Vibrio* sp. QY101(AAP45155.1), A9mT from *Vibrio* sp. JAM-A9m (BAH79131.1), AlyVOA from *Vibrio* sp. O2 (ABB36771.1), and AlyVOB from *Vibrio* sp. O2 (ABB36772.1). Identical amino acids are shaded black, whereas similar amino acids are shaded purple or light blue. Conserved regions are boxed.

The deduced amino acid sequence (190 aa) contained the sequence of KDNVMHLTFKK, which has been determined. The sequence was most homologous to the PL family 7 protein of *Vibrio* sp. CA-1004 (WP_086981048.1) with 76% identity, followed by the PL family 7 protein of *V. litoralis* (WP_027695256.1) with 75% identity, and the alginate lyase of *V. rumoiensis* (OEF27383.1) with 74% identity, indicating AlyH1 was a PL7 alginate lyase. Some PL7 alginate lyases from *Vibrio* sp., which had different substrate specificity, were selected and a sequence homology blast was performed with the identified partial amino acid sequence of AlyH1. Three highly-conserved regions R(S/N) ELR, Q(I/V) H, and YFKAG(N/V/L) Y were identified in Figure 4. Zhu et al. investigated the relationship between substrate specificity and protein sequence, and found that the poly-M-specific and poly-G-specific alginate lyases contain QVH and QIH in the conserved regions, respectively, [5]. Indeed, alginate lyases, such as AlgNJU-03 of *Vibrio* sp.NJU-03 [19] and AlyVI of *Vibrio* sp. QY101 [29], are known to preferably degrade poly-G, possessing QIH regions (Figure 4). Alginate lyase such as A9mT of *Vibrio sp*. JAM-A9m [23], AlyVOA and AlyVOB of *Vibrio* sp. O2 [27], favourably degraded poly-M, possessing QVH regions (Figure 4). AlyH1, containing the conserved region QIH, preferred poly-G as the substrate. The result was consistent with the reported alginate lyases. The identities of the partial amino acid sequence of AlyH1 to the corresponding partial amino acid sequence of alginate lyase from *Vibrio* sp.NJU-03 (ASA33933.1), *Vibrio* sp. QY101 (AAP45155.1), *Vibrio* sp. JAM-A9m (BAH79131.1), and *Vibrio* sp. O2 (ABB36771.1, ABB36772.1) were 36.36%, 21.88%, 35.32%, 17.28%, and 20.00%, respectively. Based on its relatively low sequence identities and the distinguished enzymatic properties, it might be concluded that AlyH1 was a novel enzyme. 

## 3. Materials and Methods

### 3.1. Microorganism, Media, and Culture Conditions

The alginate lyase-producing bacterial strain used in this study was isolated from rotten seaweed via an enrichment procedure. Decomposed seaweed samples were collected in Lianyungang City, China. All chemicals were of reagent grade. A selection medium using sodium alginate as the sole carbon source containing 5.0 g/L sodium alginate, 5.0 g/L (NH_4_)_2_SO_4_, 2.0 g/L K_2_HPO_4_, 30.0 g/L NaCl, 1.0 g/L MgSO_4_·7H_2_O, and 0.01 g/L FeSO_4_·7H_2_O at pH 7.0 was used for the microorganism isolation. A fermentation medium was composed of 6.0 g/L sodium alginate, 5.0 g/L tryptone, 2.5 g/L yeast extract, 25.0 g/L NaCl, 0.25 g/L KH_2_PO_4_, 0.5 g/L MgSO_4_·7H_2_O, and 0.05 g/L FeSO_4_·7H_2_O at pH 7.5. Solid medium was prepared by adding 18 g/L of agar to the above medium. Microorganisms were aerobically cultured in the 250 mL shake flasks containing 25 mL liquid medium at 28 °C and 150 rpm. 

### 3.2. Strain Isolation and Identification

The preliminary screening for the alginate-degrading microorganism was conducted as follows. First, 1 g sample of decomposed seaweed sample was suspended in 10 mL selection medium. Next, 1 mL suspension samples were transferred to 25 mL selection medium for enrichment and were cultivated at 28 °C for 48 h with continuous shaking at 150 rpm. Microorganisms that could grow in a selection medium were accumulated by subculturing on selection medium plates containing 1.8% agar for isolation until pure cultures were obtained.

The pure microorganisms were transferred to a new plate and incubated at 28 °C for 48 h, while original plates were poured with 1% CaCl_2_ solution. Strains with clear hydrolytic zones were selected and incubated aerobically in a fermentation medium under the same conditions as above for rescreening high alginate lyase activity strains. Among the isolates, the most active strain, H1, was selected for further studies.

Bacterial identification was performed based on the 16S rRNA gene sequence, which was amplified by PCR using the primers: 27F (5′-AGAGTTTGATCCTGGCTCAG-3′) and 1492R (5′-GGTTACCTTGTTACGACTT-3′). The PCR product was sequenced by Sangon Biotech (Shanghai, China). The sequence was blasted at NCBI and registered at the GeneBank database. A phylogenetic tree was built using MEGA 5.0 software (Biodesign Institute, Arizona State University, Tempe, AZ, USA) with the neighbour-joining method.

### 3.3. Purification of Alginate Lyase

Enzyme purification was performed by using an AKTA purifier (GE Healthcare, Piscataway, NJ, USA). Sodium phosphate buffer (PB, 50 mM, pH 7.5) containing 1 mM DTT was used as the standard buffer (Buffer A).

Step 1: Ammonium sulphate fractionation

The supernatant from one litre of culture supernatant were harvested via centrifugation at 8000 rpm for 20 min. Ammonium sulphate was gradually added to the crude extract with a final concentration of 30% and the mixture was stirred on ice for 1 h. The precipitate formed was removed by centrifugation (20,000× *g*, 10 min at 4 °C) and discarded. The clear supernatant obtained was subjected to increased concentration of 75% ammonium sulphate and the mixture was stirred for an additional hour on ice. The resulting precipitate was collected by centrifugation (20,000× *g*, 10 min at 4 °C) and dissolved in 40 mL Buffer A. It was then dialyzed against a large volume of Buffer A.

Step 2: Q-Sepharose chromatography

The crude enzyme solution from Step 1 was filtered through a 0.45 μm filter membrane and was then applied to a 5 mL Q-Sepharose column which had been equilibrated with Buffer A. Elution was performed with a linear gradient of NaCl (0.2–0.8 M) in Buffer A. Fractions containing alginate lyase activity were pooled and concentrated using an Amicon Ultra-4 unit (Millipore, Billerica, MA, USA).

Step 3: Superdex^TM^ 200 gel filtration chromatography

The protein solution obtained after ion exchange chromatography was placed on a gel filtration chromatography, Superdex^TM^ 200 10/300 GL column, which was pre-equilibrated with Buffer A containing 0.2 M NaCl. The enzyme was eluted with the same buffer, and AlyH1 was collected, dialyzed, and used for further study.

The protein concentration was determined by the Coomassie Brilliant Blue G-250 dye-binding method of Bradford [39] with bovine serum albumin as a standard. The molecular mass of the purified enzyme was analysed by 12.5% SDS-PAGE. The proteins were developed by Coomassie Brilliant Blue R-250 staining. 

### 3.4. Alginate Lyase Assay

Alginate lyase activity was determined by measuring the amount of released reducing sugar using the 3,5-dinitrosalicylic acid (DNS) method following Dou et al. [6] with slight modification. The reaction was initiated by adding 0.1 mL of the prepared enzyme to 0.9 mL of 1% sodium alginate (dissolved in 50 mM PB at pH 7.5). After incubation at 40 °C for 20 min, the reaction was stopped by adding 0.5 mL of DNS reagent and heating at 100 °C for 5 min. After cooling to room temperature, the activity was measured by monitoring the increased absorbance of the reaction products (reducing sugar) at 540 nm. One unit of alginate lyase activity (U) was defined as the amount of enzyme required to generate 1 mg of reducing sugar (glucose equivalent) per min. All assays were performed in triplicate.

### 3.5. Characterization of AlyH1 

#### 3.5.1. Effects of Temperature and pH on AlyH1 Activity and Stability

The effects of temperature (20–60 °C) on the enzyme were investigated at pH 7.5. The thermal stability of the enzyme was determined under the standard assay conditions after incubating purified enzyme solutions at 20–60 °C for 30 min. Meanwhile, the effects of pH on the enzyme activity were evaluated by incubating the purified enzyme in PB over the pH ranges of 6.0 to 9.5. The pH stability depended on the residual enzyme activity when the enzyme was conserved in different pH at 4 °C for 12 h in advance.

#### 3.5.2. Effects of NaCl Concentration and Metal Ions on AlyH1 Activity

The effects of NaCl on alginate lyase activity were investigated at different concentrations between 0–0.6 M under standard test conditions. The influences of different metal ions on the enzyme activity were performed by incubating the enzyme in 50 mM PB (pH 7.5) at 4 °C for 30 min in the presence of various metal compounds at a concentration of 5 mM. Further residual activities were then assayed. 

#### 3.5.3. Kinetic Parameters 

The kinetic constants of the purified enzyme were determined by measuring activity at different sodium alginate concentrations. The maximal reaction rate (*V*max) and apparent Michaelis-Menten constant (*K*m) were determined by linear regression analysis of the Lineweaver-Burke double-reciprocal plot [40].

#### 3.5.4. Substrate Specificity

To investigate the substrate specificity, 1% sodium alginate, poly-M, and poly-G were used as the substrates to determine the enzyme activity. Poly-M and poly-G were prepared via HCl-hydrolysis according to the procedure described by Gong et al. [32]. Briefly, sodium alginate (1.0 g) was dissolved in 50 mL of 1 M HCl and kept in a water bath at 90 °C for 5 h. The solution was then cooled and centrifuged at 8000× *g* for 20 min. The sediment was suspended in deionized water and adjusted to neutral pH until dissolved fully using NaOH solution. Then it was adjusted to a pH of 2.86 with HCl. After being centrifuged at 8000 × *g* for 20 min, the insoluble part (which is poly-G) was dissolved, dialyzed, and freeze-dried. Next, the supernatant was adjusted to a pH of 1.0. After being centrifuged at 8000 × *g* for 20 min, the poly-G precipitate was dissolved, dialyzed, and freeze-dried. The assay of enzyme activity was the same as previously described. 

#### 3.5.5. TLC Analysis of the Degradation Products

Thin-layer chromatography (TLC) was applied to analyse the oligosaccharides produced by the degradation of sodium alginate with the purified AlyH1. The reaction mixture containing 1 mL of 1.0% sodium alginate and 1 mL of enzyme was incubated at 30 °C for 24 h. A 5 μL aliquot of the reaction product was subjected to TLC using a solvent system of 1-butanol: acetic acid: water (3:2:2, *v*/*v*). The plate was then sprayed with 10% (*v*/*v*) sulfuric acid in ethanol and heated at 120 °C for 5 min [41].

#### 3.5.6. Determination of Partial Amino Acid Sequences of AlyH1

The identification of the internal peptide sequence of AlyH1 was carried out through mass spectrometry. The purified protein in SDS-PAGE was cut out. After destaining (100 mM NH_4_HCO_3_ and 30% acetonitrile) and washing, the dried gel pieces were treated with trypsin (50 μg/mL) in 50 mM NH_4_HCO_3_ buffer (pH 8.0) at 37 °C overnight. Considering the different molecular masses with different time-of-flight detections, these peptide fragments were analysed using an Applied Biosystem reflectron time-of-flight mass spectrometer and identified by comparison with various protein identification databases. Degenerate primers Valg1 (5-CGBTCDGARCTBCGBGMRATG-3) and Valg2 (5-RTARTTRCCBGCYTTRAARTA-3) were designed according to the conserved regions R(S/N) ELR(E/A/V) (M/T/Q) and YFKAG(N/L/A/V) Y of the PL7 alginate lyases [38] to amplify the partial sequence of the alyH1 using the genomic DNA of H1 as a template. Amplified fragments were purified and ligated with pMD19-T plasmid by T/A cloning, and transformed into *E. coli* JM109 competent cells. Finally, positive clone was sequenced by Sangon Biotech (Shanghai, China). The deduced amino sequence was analysed using DNAMAN software (Lynnon biosoft, San Ramon, CA, USA) and the BLAST tool of NCBI.

## 4. Conclusions

In this study, a new alginate lyase-producing marine bacterium was isolated and identified as *V. furnissii* H1. AlyH1 secreted by *V. furnissii* H1 was characterized as a novel PL7 alginate lyase. The enzyme showed excellent characteristics, such as a broad pH range for enzyme activity, thermal stability, and specificity for both poly-M and poly-G, and could hydrolyse alginate to produce low polymerization oligosaccharides. These characteristics indicate that AlyH1 may play a role in marine alginate degradation and carbon cycling and open potential applications for large-scale industrialization. Further works will be focused on obtaining the full length and heterologous expression of AlyH1 to further elucidate the function of the enzyme, and lay a better foundation for its practical application.

## Figures and Tables

**Table 1:** Characterization of alginate lyases from different *Vibro* sp. microorganisms.

Microorganisms	Molecular Mass (kDa)	Optimal pH/Temperature (°C)	Substrate Specificity	Reference
*V. furnissii* H1	35.8	7.5/40	Poly-M, poly-G	This study
*Vibrio* sp. SY08	33	7.6/40	Poly-M, poly-G	[17]
*Vibrio* sp. QD-5	62	8.9/35	poly-G	[18]
*Vibrio* sp. NJU-03	48.12	7.0/30	Poly-M, poly-G	[19]
*Vibrio* sp. W13	54.12	8.0/30	Poly-M, poly-G	[20]
*V. splendidus* 12B01	68.2	8.5/25	Poly-M, poly-G	[21]
	59.0	7.5/20-25	Poly-M, poly-G	[21]
	36.5	8.0/20	Poly-M, poly-G	[21]
	35.2	7.5/25	Poly-M, poly-G	[21]
	80	6.5/16	Poly-M, poly-G	[22]
	83	7.0/30	Poly-M, poly-G	[22]
	81	7.5/35	Poly-M	[22]
*Vibrio* sp. JAM-A9m	28	7.6 and 9/30	Poly-M	[23]
*Vibrio* sp. QY105	37	7.0/38	Poly-M, poly-G	[24]
*Vibrio* sp. YKW-34	60.0	7.0/40	Poly-M, poly-G	[25]
*Vibrio* sp.YWA	62.5	7.0/25	Poly-M, poly-G	[26]
*Vibrio* sp. O2	28.4	-	Poly-M	[27]
	25.2	-	Poly-M	[27]
*Vibrio* sp. 510-64	34.6	7.5/35	Poly-G	[28]
*Vibrio* sp. QY101	34	7.5/40	Poly-M, poly-G	[29]
*Vibrio* sp. AL-9	25	9.0/-	Poly-G	[30]
	31	8.0/-	Poly-M	[30]
*V. harveyi* AL-128	-	7.8/-	Poly-G	[31]

**Table 2:** Purification of AlyH1.

Purification Steps	Total Protein (mg)	Specific Activity (U/mg)	Total Activity (U)	Yield (%)	Purification (Fold)
Liquid supernatant	121.84	0.13	15.84	100.00	1.00
(NH_4_)_2_SO_4_ fractionation	79.53	0.16	12.72	80.33	1.23
Q-Sepharose chromatography	6.67	0.82	5.47	34.53	6.31
Gel filtration chromatography	0.61	2.40	1.47	9.28	18.46

## Supplementary Materials

The following are available online at http://www.mdpi.com/1660-3397/16/1/30/s1. Figure S1. Phylogenetic tree for strain H1 and related strains based on the 16S rRNA gene sequence. Numbers after the names of organisms are the accession numbers of the published sequences. The phylogenetic tree was inferred by using the neighbour-joining methods. The software MEGA 5.0 was used for analysis. Figure S2. Effects of NaCl concentration and metal ions on the activity of AlyH1. (a) Influences of the concentration of NaCl; (b) Influences of the metal ions. The enzyme activity without metal ions served as the control with the corresponding enzyme activity designated as 100%. Figure S3. Partial nucleotide sequence of the alyH1.
