# Supplementary material for: Characterization of a Novel Alginate Lyase from Marine Bacterium Vibrio furnissii H1
Source: Mar Drugs. 2018 Jan 15;16(1):30. doi: 10.3390/md16010030 (PMC5793078; doi:10.3390/md16010030)
Supplement: Supplementary file 1 [file marinedrugs-16-00030-s001.pdf]

# Supplementary Materials: Characterization of a Novel Alginate Lyase from Marine Bacterium *Vibrio furnissii* H1

**Xiaoyan Zhu, Xiangqian Li, Hao Shi, Jia Zhou, Zhongbiao Tan, Mengdi Yuan, Peng Yao, and Xiaoyan Liu**

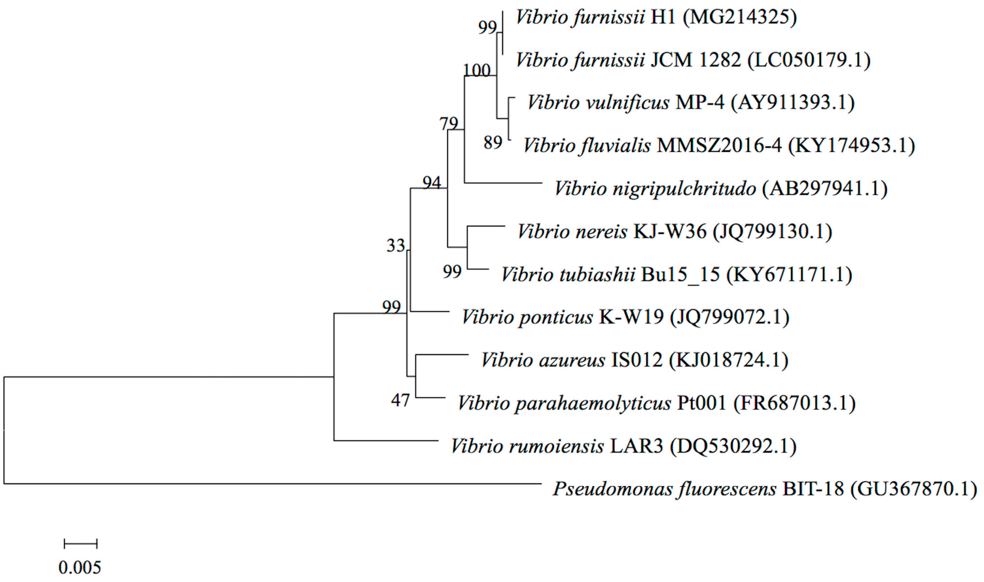


**Figure S1.** Phylogenetic tree for strain H1 and related strains based on the 16S rRNA gene sequence. Numbers after the names of organisms are the accession numbers of the published sequences. The phylogenetic tree was inferred by using the neighbour-joining methods. The software MEGA 5.0 (Biodesign Institute, Arizona State University, Tempe, AZ, USA) was used for analysis

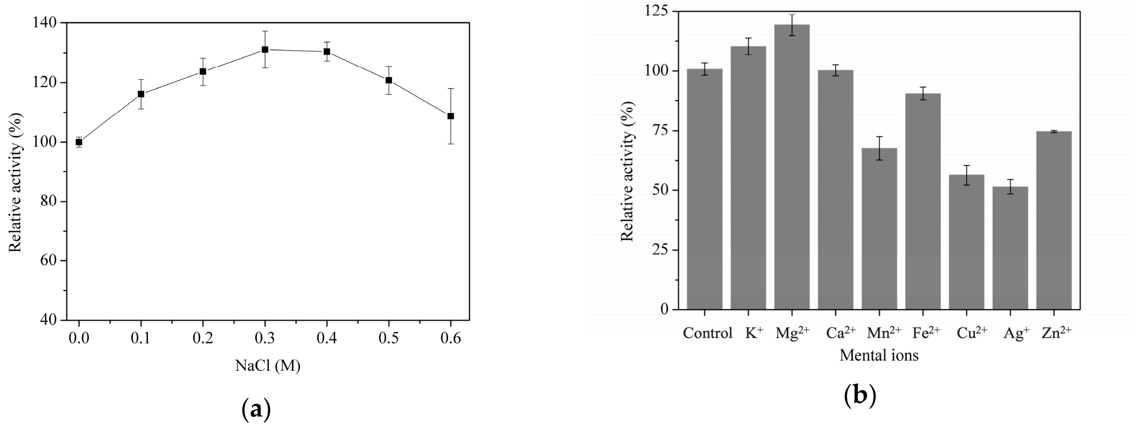


**Figure S2.** Effects of NaCl concentration and metal ions on the activity of AlyH1. (**a**) Influences of the concentration of NaCl; and (**b**) Influences of the metal ions. The enzyme activity without metal ions served as the control with the corresponding enzyme activity designated as 100%

```
1      CGGTCTGAGC TCCGGGAAAT GTTGGCAGAA GATTATGGCG AGCCTGCGAA TAACTTTGCG
61     ATTAAAGCCA ATGATAAAGT CGATGAGTAT GGTTCAGTCG GTGGCCGTTT AAGTGCAACT
121    CTTTCTGTTG ATTGGGTGAG TACGAGTGGT AATGAATCCA AATCAGGTGC TTATGCTGTG
181    ATTGTTGGTC AAATCCATGG TTCGAATAAT GAACCGTTGA AAATTGTTTA CCGCAAATTG
241    CCAAATCATG AGTTTGGTTC CTTGTCTTGG AACTATGAAA CAAACCCCGT TGAAAAAGCG
301    GATCGCACCG ATATTAAACA CGATATTTTC GGCAAAAATA AGCTCACCAG TAAAGATGCA
361    GATCCACAAG ATGGTATTCG TTTAGGTGAG GTTTTCTCTT ATGATGTGAA CGTGAAAGAT
421    AACGTCATGC ACCTGACCTT TAAGAAAAAC TTAGGTCAAG ATGATGAAGT AACCAAAACA
481    TACGATTTAG ATCTAAGTAA GCCATATCCA AATCAAGCCA AAGATACCAG CTATGCGCAA
541    GATTGGATGT ACTTTAAGGC CGGCAACTAT
```

**Figure S3.** Partial nucleotide sequence of the alyH1.
